# Primary radio(chemo)therapy for esophageal cancer in elderly patients: are efficiency and toxicity comparable with younger patients?

**DOI:** 10.1186/s40001-017-0265-x

**Published:** 2017-07-06

**Authors:** Stefan Münch, Christine Heinrich, Daniel Habermehl, Markus Oechsner, Stephanie E. Combs, Marciana-Nona Duma

**Affiliations:** 1 Department of Radiation Oncology, Klinik für RadioOnkologie und Strahlentherapie, Klinikum rechts der Isar/TU München, Ismaninger Str. 22, 81675 Munich, Germany; 2 Institute of Innovative Radiotherapy (iRT), Helmholtz Zentrum München, Ingolstädter Landstraße 1, 85764 Oberschleißheim, Germany; 3 Practice for Radiotherapy, Norbert-Kerkel-Platz 1, 83734 Hausham, Germany

## Abstract

**Purpose:**

In elderly patients with esophageal cancer (EC), esophagectomy is associated with an increased mortality, and therefore these patients are often treated with definite (chemo)radiation. The purpose of this study was to assess the toxicity and efficiency of definite radio(chemo)therapy in patients >75 years compared with definite radio(chemo)therapy in patients <75 years.

**Methods:**

32 patients >75 years were treated with definite radio(chemo)therapy for EC. We compared baseline parameters, efficiency and toxicity rates of these patients to 39 patients <75 years.

**Results:**

Patients <75 years were more likely to receive simultaneous chemotherapy, and had a lower age-adjusted Charlson comorbidity index (ACCI). 25% of elderly patients were treated in palliative intent. There was no significant difference in progression-free survival between patient groups. No significant differences were seen for overall survival (15.7 months vs. 19.9 months; *p* = 0.102) and progression-free survival (10.5 months vs. 9.2 months, *p* = 0.470) between older patients treated with curative intent and younger patients. In addition, there were no significant differences for dysphagia and hematological side effects between elderly patients and younger patients.

**Conclusion:**

Definite (chemo)radiation is a feasible therapy for elderly patients. OS and PFS in elderly patients with a curative treatment approach are comparable to younger patients and it is not associated with higher toxicity rates.

## Background

Every year, more than 450,000 new cases of esophageal cancer (EC) are diagnosed, and an increase of 140% is predicted over the next ten years [1, 2]. With a median age of 67 years at diagnosis and 30% of patients being 75 years or older, EC is a cancer of the elderly. Due to demographic changes, this is sure to become a challenge in the near future [3].

In recent years, studies demonstrated the advantage of neoadjuvant chemoradiation (nCRT) over surgery alone, and therefore, multimodal therapy was established as the standard treatment for patients suitable for surgery [4–6].

However, for elder patients (>70–80 years) esophagectomy is associated with an increased mortality and lower overall survival than for younger patients [7–10]. Therefore, the rate of surgery is significant lower among these patients [11, 12]. Instead of surgery, elder patients are often treated with primary radio(chemo)therapy, but, because they are often excluded from clinical trials, different regimes are used for these patients [13–16]. Nonetheless, most of the available studies demonstrate the feasibility of definitive radio(chemo)therapy in elderly patients with acceptable survival rates and treatment-related toxicities [13, 14, 17, 18].

Whether the therapy regimes from younger patients can be applied to elder patients remains controversial, and data are scarcely available. Takeuchi et al. [15] compared primary radio(chemo)therapy in 33 patients older than 71 years with 145 patients younger than 70 years. Therapy consisted of radiotherapy up to 60 Gy and simultaneous chemotherapy with 5-fluorouracil and Cisplatin. An age of >71 years was associated with a higher rate of toxicities and an inferior overall survival. After a median follow-up of 57 months, median survival time was 14.7 months for patients >71 years and 35.1 months for patients <70 years. For elderly patients, there was also an increased risk of ≥III° leukopenia or anemia.

The aim of our study was to compare the efficiencies and side effects in patients older than 75 years with the outcomes of younger patients treated with definitive radio(chemo)therapy for esophageal cancer.

## Methods

Between 1999 and 2014, 32 elderly patients (>75 years) were treated with definite radio(chemo)therapy for EC at our institution. For the control group, we also analyzed 39 younger patients who received definite (chemo)radiation between 2008 and 2014. Efficiency and toxicity was then retrospectively compared between both patient groups. Inclusion criteria were a histologically proven EC and definitive or palliative radio(chemo)therapy without further surgery.

Medical records and follow-up data were used to analyze survival and outcomes of patients. Follow-up of all patients was done according to our institutional guidelines beginning 6–8 weeks after treatment and every 3 months thereafter for 2 years and then every 6 months for another 2 years. Follow-up examinations always consisted of a clinical examination and an esophago-gastro-duodenoscopy, and, case by case, of a thoracic computer tomography. In case of missing information, the database of the Munich cancer registry was checked for information about survival or recurrence.

Comorbidities of patients were analyzed based on the “*age*-*adjusted Charlson comorbidity index*” (ACCI) [19], which can be used. e.g., as a predictor of early postoperative complications after esophagectomy [20]. The performance status was defined according to the Eastern Cooperative Oncology Group (ECOG) definition. Hematological side effects and dysphagia were retrospectively classified according to the common terminology criteria for adverse events (CTCAE) v4.03 after the review of medical records.

Staging by computer tomography was more often available for younger patients than for elderly patients (100% vs. 88%; *p* = 0.037). While 95% of younger patients were also staged with positron-emission tomography (PET), this was only available for 50% of elderly patients (*p* < 0.001). The significant difference in PET–CT staging is due to the recruitment period of elderly patients. PET/CT staging had not been established as a standard when several of these patients were treated.

For treatment planning, the GTV (primary tumor and lymph node metastases) were delineated using all available diagnostic information (esophago-gastro-duodenoscopy, endosonographic ultrasound, and PET). While the primary tumor and locoregional lymph node metastases were irradiated in all patients, the elective lymphatic pathways were irradiated in all curative treated patients.

Radiotherapy was done by means of 3D-conformal radiotherapy, intensity-modulated radiation therapy (IMRT), or volumetric-modulated arc-therapy (VMAT). As different radiotherapy fractionations were used for the older patients (1.8–3 Gy single doses to total doses of 7–65 Gy) in this study, we report the cumulative 2 Gy equivalent dose (EQD2). The EQD2 was calculated with an α/β ratio of 10.

The distribution of quantitative data is described by median and interquartile range (IQR). Likewise, qualitative data are presented as absolute and relative frequencies. Corresponding statistical hypothesis analyses are performed using Wilcoxon–Mann–Whitney-U tests and Fisher’s exact test, respectively. Overall survival (OS) and progression-free-survival (PFS) where calculated from results at the end of the therapy. The comparison between patient groups was done by the log-rank-tests. All statistical analyses were conducted in an exploratory manner on two-sided 5% significance levels using the software *PASW Statistics 18 version 18.0.0.*

## Results

The median age of the elderly patients’ was 82 years (IQR 79–84 years), whereas the median age of the younger patients’ was 66 years (IQR 59–72 years; *p* = 1.000). The ECOG status of patients >75 years was lower than that of the younger patients. 20% of elderly patients had an ECOG status of “0.” 73% had a status of “1.” and 7% had a status of “2,” whereas almost 73% of patients <75 years had an ECOG performance status of “0,” and 27% had a status of “1” (*p* < 0.001). Elderly patients also had more comorbidities with a higher median ACCI than had the younger patients (7 vs. 3; *p* < 0,001). All (100%) of the younger patients had a squamous cell carcinoma (SCC) of the esophagus. In contrast to this, 19% of elderly patients were diagnosed with adenocarcinoma and 81% with SCC. While no significant differences between the patient groups were observed for the other baseline characteristics such as sex, TNM classification, UICC-stage, grading, tumor length, and localization, significantly more elderly patients were treated in palliative intent (25% vs. 0%; *p* = 0.001) and also were less likely to receive any kind of chemotherapy (40.6% vs. 82.1%; *p* < 0.001). Compared with elderly patients with curative intent, younger patients were more likely to have adenocarcinoma (21% vs. 0%; *p* = 0.006), had a higher ACCI (7 vs. 4; *p* < 0.001), and had a higher ECOG performance status (*p* < 0.001). In addition, younger patients were also more likely to receive simultaneous chemotherapy (82% vs. 42%; *p* = 0.002) (Table 1).

**Table 1:** Patients’ characteristics

Patient characteristics	Age >75 years only curative intent (*n* = 24)		Age >75 years only palliative intent (*n* = 8)		Age ≤75 years (*n* = 39)		*p* value^a^
	%	Median	%	Median	%	Median	
		(IQR 25–75)		(IQR 25–75)		(IQR 25–75)	
Sex							1.000
Male (%)	66.7		62.5		66.7		
Age in years		81		83		66	<0.001
		(78–85)		(79–84)		(59–72)	
ECOG							<0.001
0	17.4		28.6		73.0		
1	78.3		57.1		27.0		
2	4.3		14.3		0.0		
ACCI		7		8		4	<0.001
		(6–8)		(6–9)		(4–5)	
Histology							0.006
SCC (%)	79.2		66.7		100.0		
Adenocarcinoma (%)	20.8		33.3		0.0		
Primary tumor extension							0.353
cT1 (%)	12.5		0		8.1		
cT2 (%)	4.2		37.5		5.4		
cT3 (%)	83.3		62.5		75.7		
cT4 (%)	0		0		10.8		
Lymph node extension							0.721
cN+ (%)	83.3		100		87.2		
Distant metastasis							0.268
M1 (%)	0		25		8.3		
UICC-stage							0.239
IA (%)	8.3		0		0		
IIA (%)	8.3		0		11.1		
IIB (%)	8.3		37.5		11.1		
IIIA (%)	75		50		61,1		
IIIC (%)	0		0		8.3		
IV (%)	0		12.5		8.3		
Grading							0.492
G1 (%)	4.2		0		0.0		
G2 (%)	50		25		57.1		
G3 (%)	45.8		75		42.9		
Tumor localization							0.195
Upper third (%)	26.1		25		42.1		
Middle third (%)	43.5		50		23.7		
Lower third (%)	30.4		25		34.2		
Tumor length (centimeter)		5		7		5	0.245
		(3–7)		(5–9)		(5–7)	
Discontinuation of treatment (%)	0.0		60		0.0		
Simultaneous chemotherapy (%)	41.7		37.5		82.1		0.002
EQD2 (Gy)		54		40		53	0.417
		(53–55)		(19–46)		(53–54)	
Overall dose (Gy)		54		38		54	0.860
		(54–56)		(20–45)		(54–54)	
Daily dose (Gy)		1.8		2.0		1.8	0.127
		(1.8–2)		(1.8–2.5)		(1.8–1.8)	

The most common side effect was dysphagia. All of the elderly patients and more than 84% of the younger patients described some kind of dysphagia. No significant differences were seen for acute hematological toxicities and dysphagia when comparing younger patients with all elderly patients, as seen in the case when younger patients are compared with elderly patients with only curative intent (Tables 2, 3)

*SCC* squamous cell carcinoma, *EQD2* 2 Gy equivalent dose, *ECOG* Eastern Cooperative Oncology Group performance status, *ACCI* age-adjusted Charlson comorbidity Index

^a^ *p* value refers to the comparison between younger patients and elderly patients with curative intent

**Table 2:** Adverse events for all patients

Adverse events	Age >75 years (*n* = 32)	Age ≤75 years (*n* = 39)	*p* value
Dyspahgia (CTCAE)			0.132
0° (%)	0.0	15.4	
I° (%)	50.0	43.6	
II° (%)	28.1	20.5	
III° (%)	21.9	17.9	
IV (%)	0.0	2.6	
Leukopenia (CTCAE)			0.105
0° (%)	34.4	26.3	
I° (%)	18.7	18.4	
II° (%)	37.5	26.3	
III° (%)	6.3	29.0	
IV (%)	3.1	0.0	
Anemia (CTCAE)			0.157
0° (%)	3.1	17.9	
I° (%)	56.3	38.5	
II° (%)	37.5	41.0	
III° (%)	3.1	2.6	
Thrombocytopenia (CTCAE)			0.795
0° (%)	31.3	34.3	
I° (%)	50.0	51.4	
II° (%)	18.7	11.4	
III° (%)	0.0	0.0	
IV (%)	0.0	2.9

**Table 3:** Adverse events for patients with curative intent only

Adverse events	Age >75 years (*n* = 24)	Age ≤75 years (*n* = 39)	*p* value
Dyspahgia (CTCAE)			0.187
0° (%)	0.0	15.4	
I° (%)	54.2	43.6	
II° (%)	33.3	20.5	
III° (%)	12.5	17.9	
IV (%)	0.0	2.6	
Leukopenia (CTCAE)			0.157
0° (%)	33.3	26.3	
I° (%)	12.5	18.4	
II° (%)	41.7	26.3	
III° (%)	8.3	29.0	
IV (%)	4.2	0.0	
Anemia (CTCAE)			0.061
0° (%)	0.0	17.9	
I° (%)	62.5	38.5	
II° (%)	33.3	41.0	
III° (%)	4.2	2.6	
Thrombocytopenia (CTCAE)			0.958
0° (%)	29.2	34.3	
I° (%)	58.3	51.4	
II° (%)	12.5	11.4	
III° (%)	0.0	0.0	
IV (%)	0.0	2.9	

Of the 31 younger patients who received simultaneous chemotherapy, 29 (94%) received a combination of cisplatinum (20 mg/m^2^/day; days 1–5 and days 29–33) and 5-fluorouracil (500 mg/m^2^/day; days 1–5 and days 29–33). In two other younger patients (6%), simultaneous chemotherapy was performed with 5-fluorouracil only (250 mg/m^2^/day, continuous).

Of the 13 older patients who received simultaneous chemotherapy, eight patients (62%) received a combination of cisplatinum (20 mg/m^2^/day; days 1–5 and days 29–33) and 5-fluorouracil (500 mg/m^2^/day;, days 1–5 and days 29–33). Simultaneous chemotherapy with 5-fluorouracil (250 mg/m^2^/day; continuous) only or cisplatinum (40 mg/m^2^/day; weekly) only was performed in two patients and two patients, respectively. In one another patient, chemotherapy was performed with carboplatinum (area under the curve 5, week 1 + 5).

One of the younger patients and one elderly patient each received additional induction chemotherapy with 2 cycles of cisplatinum and 5-fluorouracil.

On comparing the side effects between younger patients and older patients who received simultaneous chemoradiation (*n* = 10), there was no significant differences for dysphagia, anemia, and thrombocytopenia. In contrast, I°–IV° leukopenia was seen in 0, 70, 10, and 10% of older patients and 19, 29, 35, and 0% of younger patients, respectively (*p* = 0.037).

### Local control

48.6% of younger patients and 41.6% of older patients with curative intent had a local relapse. However, this difference was not statistically significant (*p* = 0.611).

### Survival

Median follow-up was 15.4 (IQR 5.3–23.2) months for elderly patients with curative intent and 18.3 (IQR 6.8–38.2) months for corresponding younger patients.

**Fig. 1:**
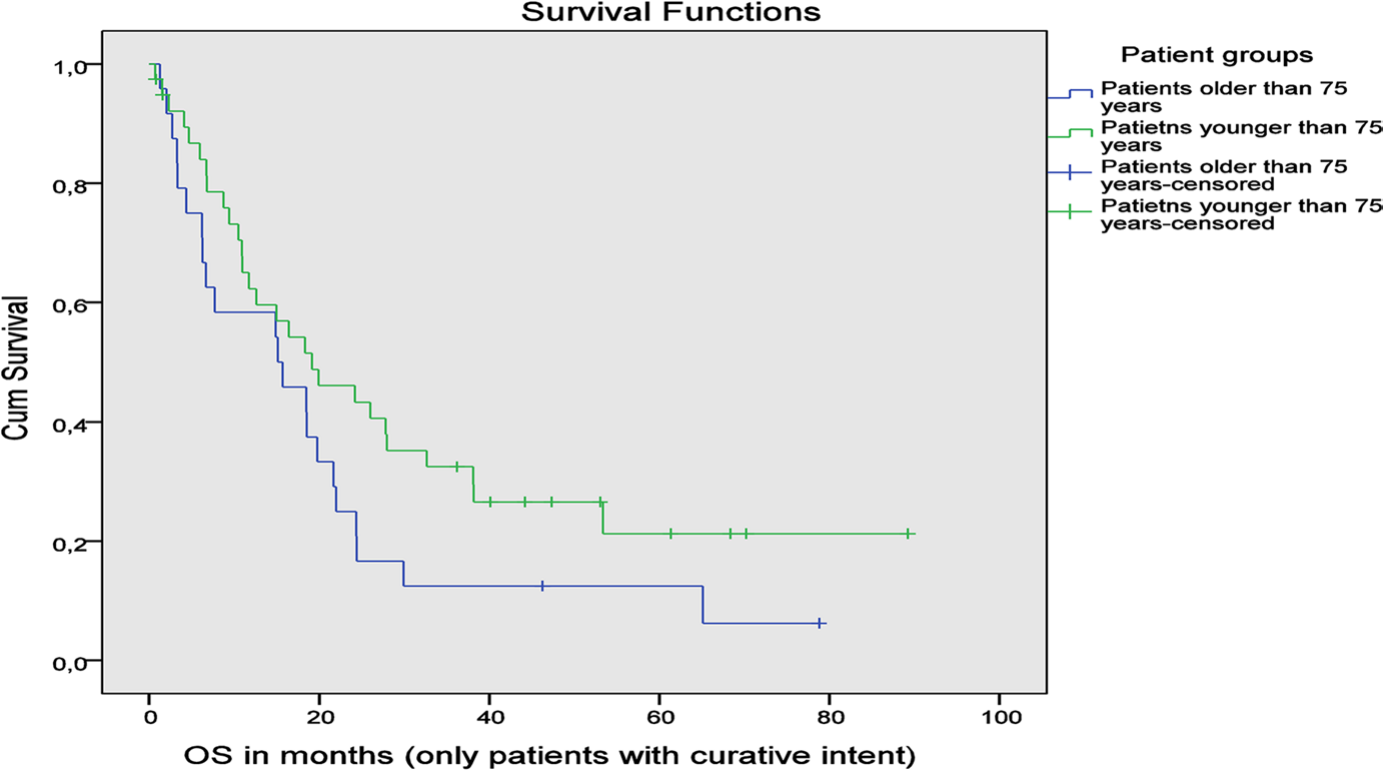
Overall survival of patients treated with a curative treatment approach

Median overall survival (OS) was 7.7 months for elderly patients and 19.9 months for younger patients (*p* = 0.016). However, there was no significant difference for OS in younger patients compared with elderly patients with a curative treatment intent (15.7 months vs. 19.9 months; *p* = 0.102) (Fig. 1). Elderly patients treated with a palliative treatment intent had a median OS of 2.7 months.

**Fig. 2:**
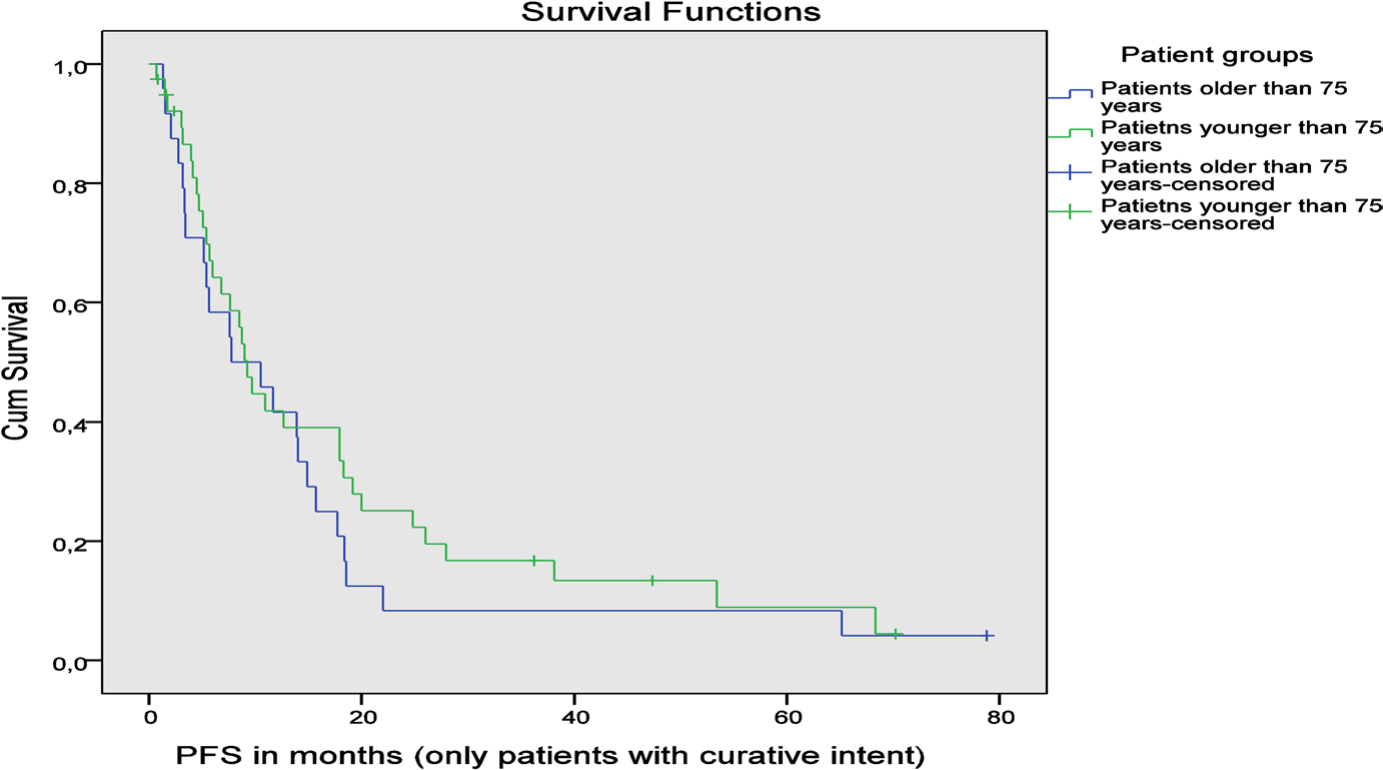
Progression-free survival of patients treated with a curative treatment approach

No significant differences in PFS were seen between younger patients and all older patients (9.2 months vs. 7.6 months; *p* = 0.203), or when comparing younger patients with older patients treated with curative intent (9.2 months vs. 10.5 months; *p* = 0.470) (Fig. 2).

## Discussion

In this study, we evaluated the toxicities and the outcome of definite radio(chemo)therapy for esophageal cancer in patients older than 75 years compared with patients younger than 75 years. Our study has some limitations. The most important is it is retrospective in nature with all the inherent problems concerning follow-up data. One way that we tried to compensate for the missing of follow-up data was the use of the Munich cancer registry survival data.

Despite these limitations, our results are comparable to those available in the literature. Two studies by Minsky et al. [21] and Conroy et al. [22] investigated the efficiencies of (curative) radio(chemo)therapy in patients with esophageal cancer. In 109 patients with a median age of 64 years, who were treated with 50.4 Gy and a concurrent chemotherapy with cisplatinum and 5-fluorouracil median overall survival was 18.1 months [21]. Another group of 133 patients with a mean age of 60 years, treated with 50 Gy and concurrent chemotherapy with cisplatinum and 5-fluorouracil, had a median overall survival of 17.5 months and a progression-free survival of 9.4 months [22]. The results are similar to those of our younger patient group.

When excluding patients with palliative treatment intent, OS and PFS of elderly patients in our study were 15.7 months and 9.2 months, respectively. These results correspond very well to the results mentioned above and indicate that radio(chemo)therapy is as effective in patients >75 years as in younger patients.

Previous studies about curative radio(chemo)therapy demonstrated the feasibility of this in elderly patients [14, 18]. In a study by Rochigneux et al. [18], 58 patients with a median age of 77.9 years were treated with definite radio(chemo)therapy and a mean radiation dose of 50.9 Gy. OS and PFS were 14.5 and 9.6 months, respectively. When we compare only the elderly patients treated in our institution in a curative intent, there was just a small difference in OS (15.7 vs. 14.5 months) and PFS (11.8 vs. 9.2 months) relative to the Rochigneux et al. study. In another study by Tougeron et al. [14], 109 patients older than 70 years with a mean age of 74.4 years were treated with definite radio(chemo)therapy for EC. Median OS and PFS were 15.3 and 8.2 months, respectively.

One fairly recent prospective study analyzed the efficacy and toxicity of palliative chemoradiotherapy in 25 patients with advanced/metastatic esophageal cancer [23]. The palliation aim was the reduction of dysphagia. All patients were younger than 75 years and received concomitant chemoradiation. The overall survival in this study was 7 months. Our study had an OS of approximately 3 months in the palliative treatment group of elderly patients. However, it is to be noted that many of our elderly patients had more comorbidities, a higher ACCI score, and a lower ECOG-score than the younger patients in our group. It could be hypothesized that the younger age—probably paired with a better ACCI and ECOG scores—and the combination with systemic treatment in the Akl et al. study explains the difference in OS.

No significant differences of toxicities were found between younger patients and elder patients, neither for the whole group nor for the elder patients treated in curative intent.

Compared to the results by Rochigneux et al. [18], there is a similar rate of grade 3–4 dysphagia in elderly patients (21.9% vs. 19.1%).

Younger patients were more often staged with CT and or PET/CT than elderly patients, which might cause a bias. However, in absolute numbers, CT staging was only missing in three elderly patients treated with curative intent and one elderly patient with palliative intent. There was much larger difference regarding the availability of PET between older patients and younger patients (95% vs. 50%).The significant difference in PET–CT staging is due to the recruitment period of elderly patients. PET/CT staging was not established as a standard when several of these patients were treated. However, as PET–CT might point out the metastatic patients, who will not undergo primary radiochemotherapy, the lack thereof might only make the results worse in the elderly group.

## Conclusion

Definitive radio(chemo)therapy for esophageal cancer is a feasibly therapy for elderly (in our study with a median age of 82 years) patients. Despite the fact that elderly patients have more comorbidities, a lower ECOG-score and receive less chemotherapy, there are just negligible differences in OS and PFS when stratifying for treatment approach. In elderly patients with a curative treatment approach, results are comparable with younger patients, without an increased risk for radiotherapy induced dysphagia or hematological side effects.

## Abbreviations

EC: esophageal cancer
ACCI: adjusted Charlson comorbidity index
nCRT: neoadjuvant chemoradiation
ECOG: Eastern Cooperative Oncology Group
CTCAE: common terminology criteria for adverse events
IMRT: intensity-modulated radiation therapy
VMAT: volumetric-modulated arc-therapy
EQD2: 2 Gy equivalent dose
IQR: interquartile range
OS: overall survival
PFS: progression-free survival
SCC: squamous cell carcinoma
